# Exploring Experiences of Delayed Prescribing and Symptomatic Treatment for Urinary Tract Infections among General Practitioners and Patients in Ambulatory Care: A Qualitative Study

**DOI:** 10.3390/antibiotics5030027

**Published:** 2016-08-15

**Authors:** Sinead Duane, Paula Beatty, Andrew W. Murphy, Akke Vellinga

**Affiliations:** 1 Discipline of General Practice, School of Medicine, National University of Ireland Galway, Galway, Ireland; p.beatty2@nuigalway.ie (P.B.); andrew.murphy@nuigalway.ie (A.W.M.); akke.vellinga@nuigalway.ie (A.V.); 2 Discipline of Bacteriology, School of Medicine, National University of Ireland Galway, Galway, Ireland

**Keywords:** Urinary tract infection, symptomatic treatment, delayed prescribing, antibiotic treatment, general practice, back-up prescribing

## Abstract

“Delayed or back up” antibiotic prescriptions and “symptomatic” treatment may help to reduce inappropriate antibiotic prescribing for Urinary Tract Infections (UTI) in the future. However, more research needs to be conducted in this area before these strategies can be readily promoted in practice. This study explores General Practitioner (GP) and patient attitudes and experiences regarding the use of delayed or back-up antibiotic and symptomatic treatment for UTI. Qualitative face to face interviews with General Practitioners (n = 7) from one urban and one rural practice and telephone interviews with UTI patients (n = 14) from a rural practice were undertaken. Interviews were analysed using framework analysis. GPs believe that antibiotics are necessary when treating UTI. There was little consensus amongst GPs regarding the role of delayed prescribing or symptomatic treatment for UTI. Delayed prescribing may be considered for patients with low grade symptoms and a negative dipstick test. Patients had limited experience of delayed prescribing for UTI. Half indicated they would be satisfied with a delayed prescription the other half would question it. A fear of missing a serious illness was a significant barrier to symptomatic treatment for both GP and patient. The findings of this research provide insight into antibiotic prescribing practices in general practice. It also highlights the need for further empirical research into the effectiveness of alternative treatment strategies such as symptomatic treatment of UTI before such strategies can be readily adopted in practice.

## 1. Introduction

With sustained spread of antibiotic resistance (ABR) and its increasing threat to public health, it is necessary to review antibiotic prescribing practices for infections. Recent NICE guidance (National Institute of Health and Care Excellence) published in the United Kingdom promote “delayed or back up” antibiotic prescriptions and “self care” with over the counter preparations when the infection is likely to be self-limiting [1]. Delayed prescribing strategies has been highlighted as an effective method of reducing ABR for acute respiratory infection [2]. In delaying a prescription the General Practitioner (GP) instructs the patient only to take the medication if there is no improvement in their condition or if their symptoms worsen [3]. While there are variations in how a delayed prescription is implemented, with the delay varying from one to seven days, it is designed to allow for the natural resolution of the illness during the specified time. Using this approach, inappropriate antibiotic consumption can potentially be greatly reduced [4,5]. The majority of evidence evaluating delayed prescribing refers to upper respiratory tract infections (URTI), otitis media and sore throats [2,4,5,6,7,8].

There is however an opportunity to adopt a delayed prescription strategy when treating a suspected uncomplicated urinary tract infection (UTI) in an effort to reduce inappropriate antibiotic prescriptions. For instance, though national treatment guidelines recommend empirical antibiotics, a recent study found that only 21% of patients with UTI symptoms had bacteriological confirmation of infection [9]. There is also emerging evidence that delayed prescribing in the treatment of UTI is becoming more acceptable in practice [10,11] and patients’ attitudes, behaviours and expectations towards consuming antibiotics are changing. In addition to decreased antibiotic consumption, patients who received a delayed prescription for UTI were less likely to re-attend for a further consultation [12].

In addition to delayed prescribing, a number of randomised control trials (RCT) are currently evaluating symptomatic treatment of UTI (as a variation of delayed prescribing) in general practice or ambulatory care. Symptomatic treatment differs from delayed or back up treatment in that the patient is treated with pain relief only, for example ibuprofen. Studies comparing antibiotic treatment of UTI with symptomatic treatment showed better outcomes in symptom severity. Bleidorn et al. (2010) found that ibuprofen was equally as effective as ciprofloxacin in terms of the symptomatic control of UTI [13]. However, the sample size for this pilot study was small. The subsequent full trial conducted in Germany showed that two thirds of women who received symptomatic treatment recovered without any antibiotics, however, their burden of symptoms was longer. The authors of the German RCT concluded that symptomatic treatment should be used as part of a shared decision making process with a delayed prescription with women who are experiencing mild to moderate UTI symptoms [14]. To date no studies have examined the factors that influence the GP’s decision to use immediate prescription, delayed prescription or symptomatic prescription for a UTI, or how a patient feels about these treatment options in ambulatory care. The aim of this feasibility study is to explore GP and patient attitudes and experiences regarding the use of delayed antibiotic and symptomatic treatment for UTI in ambulatory care. The results of this feasibility study will help identify how widespread delayed and symptomatic treatment are in practice and will inform the design of a broader RCT in the future.

## 2. Methods

### 2.1. Participant Selection and Procedure

Purposeful non-probability sampling was used to recruit participants between August and September 2014. In total, seven face to face interviews were conducted with GPs (n = 3 male and n = 4 females). All GPs from one rural practice (n = 6) were invited to participate. One declined, another GP was excluded from analysis as the dictaphone failed to record. This practice was selected as the researchers had access to it. UTI is a very common illness and all GPs should have experience treating patients with this illness. Three GPs were invited to participate from an urban practice. Both practices were selected as they had a mixture of male and female and had GPs with a range of levels of experience. Both practices welcomed private fee paying patients and public GMS (General Medical Services) patients. Within Ireland, private fee paying patients pay between €40 and €60 to consult their GP while GMS patients receive free health care with a co-payment of approximately €1.50 per prescription. Approximately 30% of the Irish population are entitled to the GMS scheme [15]. Payment of consultations and re-consultations may be one factor that influences the expected outcome of the consultation however others also exist.

Telephone interviews were conducted with female UTI patients from one rural practice. It is acknowledged that this is a limitation of the study however, the researchers had difficulty accessing patients from other practices within the time constraints of this project. Eligible patients were adult females who presented to their GP with symptoms suggestive of an UTI (dysuria, frequency, supra pubic pain, etc.) and consented to a telephone interview. Exclusion criteria included fever, known abnormality of the urinary tract, suspected pyelonephritis or insufficient comprehension of English. GPs provided the patient with an information leaflet explaining the purpose for the study and asked for consent to pass on their contact information (telephone number and name) to the researchers.

Nineteen patients were recruited to the study and a total of fourteen patient telephone interviews were completed. Five patients were excluded from the study after they consented to be contacted. One patient was difficult to comprehend due to unrelated health issues and another patient provided incorrect contact information. After several unsuccessful attempts to contact three patients they were also excluded.

Recruitment for this study continued until no new themes emerged from the interviews.

Ethical approval for the study was obtained from the Ethical committee of the Irish college of General Practitioners (1st August 2014) (ICE/2011/10).

### 2.2. The Interview

Semi-structured topic guides were used to achieve both flexibility of conversation and depth of content. A literature review was conducted to structure topic guides and the same researcher conducted all interviews. We employed an iterative process to ensure that any new topics emerging were fully explored.

The GP topic guide examined the factors that influence the GP’s decision to use immediate prescription, delayed prescription or symptomatic treatment for a UTI. Patient interviews focused on what behaviours impacted on GP decision making and their perception of the treatment received. Table 1 outlines the key sections discussed within the interviews and a sample of the questions asked within each section.

### 2.3. Analysis

Framework analysis was used as a matrix to organise and analyse the themes. Framework analysis was chosen as it allowed the researchers (PB and SD) to compare the data across cases (interviewees) as well as within cases. The seven step procedure for applying Framework analysis was followed [16]. Each interview was transcribed verbatim. Three transcripts were then compared against the audio recordings and found to be accurate. All transcripts were read independently by two researchers in a process of familiarisation. The researchers recorded initial impressions of the interviews on the transcripts. The transcripts were then open coded and labelled which allowed for the identification of interesting segments related to our research objectives. Once the coding process was complete the researchers met to discuss the development of the analytical framework which emerged from the open codes and labels. A set of codes (themes) were identified and defined and these were used to undertake an in-depth analysis of the transcripts. PB applied the analytical framework within excel and SD analysed a subsample to ensure rigour within the analysis process. The GP framework contained 6 broad themes, with subthemes ranging from 2–8. The patient framework contained 4 broad themes with subthemes ranging from 2–7. Table 2 provides an example of one of the themes and sub-themes used within the analysis.

## 3. Results

### 3.1. GP Results

#### GPs Attitudes towards Delayed Prescribing in Practice

Every GP has used delayed prescribing in various circumstances. Often when the GP feels pressurized by the patient or when they feel a patient is presenting early. Most commonly, a delayed prescription was given for symptoms suggestive of upper respiratory tract infections.

“Generally it’s a parent of a child that feels that the child needs an antibiotic but the child seems reasonably well and all parameters are within normal limits. It’s probably a learnt response from a previous inappropriate prescription.”

(Young Female rural GP)

The main reason a GP issues a delayed prescription is to ensure the patient will not re-consult. The GP usually has no means of knowing whether the patient consumed the antibiotic unless they ask them in a follow up consultation.

“Delayed prescriptions are issued as a favour to the patient so they don’t have to come back and pay again. It’s a protective mechanism of what if.”

(GP 1, Male)

**Figure 1:**
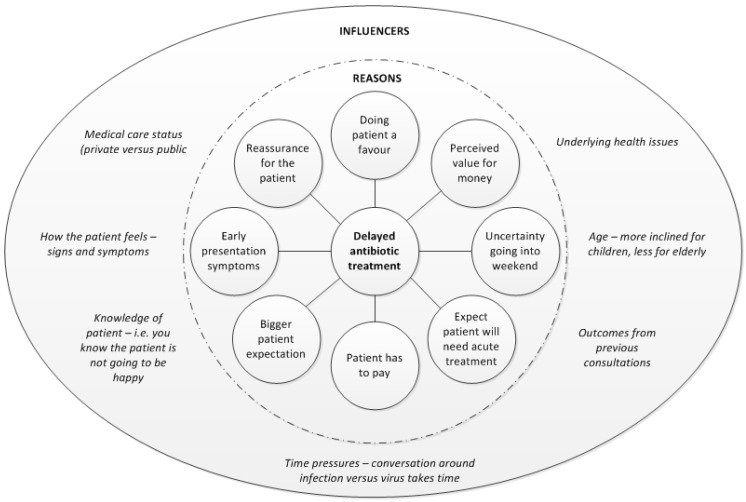
Motivators and Influences on GPs decision to provide a delayed antibiotic prescription.

Figure 1 summarises the main motivators and influences on a GPs decision to issue a delayed prescription. Any combination could result in a delayed prescription. Children were more likely to receive a delayed prescription than elderly patients who were deemed higher risk.

A delayed prescription was usually verbally explained to the patient with the recommendation only to begin antibiotic treatment if symptoms worsen. Only one GP, wrote “delayed” on the prescription.

“I have only recently started writing the delay on the prescription. I do it if I feel the patient might be more inclined to use it sooner than I would think necessary.”

(Female rural GP)

The majority of GPs recorded the delayed prescription in the patient notes and verbally followed up with them at their next consultation. The time suggested to delay a prescription ranged from 24 h to 72 h.

### 3.2. Delayed Prescribing for UTI

Most GPs believed that once they were confident the patient has a UTI, by listening to their symptoms and a positive dipstick test, the patient will ‘need’ antibiotic treatment.

“I don’t see the reason to wait for it to be honest. I don’t see why the patient should be in pain for three days just so you can be factually correct.”

(Male rural GP)

“If it’s a confirmed UTI I will always treat.”

(Young female GP)

Two GPs also indicated that patients with past experience of a UTI will generally expect an antibiotic.

“If a patient has a past history of the infection they will expect an antibiotic. Some of them will come in and say I have cystitis and I need an antibiotic…”

(Male urban GP)

GPs may consider delaying antibiotic treatment if the patient was symptomatic and the dipstick negative. In this scenario, the GP may be uncertain that the patient has a UTI but with a delayed antibiotic prescription, treatment is available if symptoms worsen and the patient decides the treatment is necessary.

If a patient has symptoms of a UTI, their GP may also advise to take pain relief, something GPs feel patients may not have considered.

### 3.3. Symptomatic Treatment for UTI

GPs said they rarely use symptomatic treatment for UTI but would consider symptomatic treatment if the patient had vague UTI symptoms. GPs agree that it is not appropriate to treat patients with antibiotics if there is a negative dipstick. Fluids and pain relief can help to manage symptoms, which patients often forget.

“I think generally people are unaware of taking pain relief for cystitis. Antibiotics don’t treat pain.”

(Male rural GP)

The concern with recommending symptomatic treatment only is that the symptoms become more severe and dissatisfied patients re-consult.

“The fear is always there that you miss something and you don’t treat it properly and somebody gets quite sick. Then you feel a bit silly for just giving them a bit of Brufen.”

(GP 1)

“For GPs the big con is that they might come back to the surgery the following day if you don’t prescribe an antibiotic.”

(Female rural GP)

The positive aspect of symptomatic treatment is that patients feel better and you are reducing our reliance on antibiotics.

“I think it works. You get people on board and you get less recurrent UTI. You get less people looking for antibiotic treatment. The patients are happy because they get some relief of symptoms.”

(Female urban GP)

### 3.4. Patient Results

#### 3.4.1. Current UTI Experience

The majority of patients attended their GP by day four of their symptoms (Table 3).

Patients attended the GP because they felt their symptoms were persistent and severe.

“I was just worn out and I felt I had to put a stop to it.”

(Patient 3)

All patients indicated that nothing would prevent them attending their GP with these symptoms. One patient specified that though “cost is a consideration” (Patient 6) it would not prevent her attending if she felt the symptoms were severe enough.

Some patients tried alternative self care methods to manage their symptoms before consulting their GP. These measures included drinking water or cranberry juice, taking over the counter preparations or taking pain relief. Some also reported drinking or washing with baking soda.

The majority of patients expected to be given an antibiotic prescription whilst a quarter indicated they only consulted the GP to rule out a possible infection. One patients consulted their GP to relieve their symptoms, another indicated they had no particular expectation at all on visiting her GP with symptoms of a UTI.

Ten out of the fourteen patients were prescribed an antibiotic to treat their UTI. Most were satisfied with this outcome as they felt they “needed” it at the time, they had received one before and they associated antibiotic treatment with symptom relief.

“I felt great because I didn’t have pain in my lower back and it was more bearable. I found the antibiotics killed the pain in my lower back and I only needed to take one or two painkillers.”

(Patient 12)

One patient however, felt she had sought antibiotic treatment too soon.

“I feel if I stayed at home a little bit more and drank more cranberry juice I might get better without antibiotics.”

(Patient 13)

Two patients were prescribed an antibiotic but did not take them as their symptoms improved. Only one patient was prescribed delayed treatment with recommended pain relief, she was satisfied with this outcome.

“I was very happy with this because I’m not one for taking antibiotics if I can avoid it at all.”

(Patient 6)

#### 3.4.2. Delayed Antibiotic Prescribing for UTI

Only two of the patients interviewed had experience with delayed treatment for UTI. One eventually started treatment the other did not. Patients without experience with delayed treatment for UTI had mixed attitudes. Half of patients would follow the GPs’ advice as they trusted their GP and viewed a delayed prescription as a safety net if symptoms persisted.

“I really am an anti-tablet person. I wouldn’t have taken them unless I desperately needed them.”

(Patient 12)

One of these patients additionally stated her acceptance would depend on how much “pain” she was in at the time.

The other half of patients felt apprehensive about delaying treatment as they perceived their symptoms to be severe enough to warrant immediate antibiotic treatment and they would not have consulted unless they needed treatment.

“I would have been a bit worried because I wouldn’t have gone to the GP unless it was very severe at the time.”

(Patient 5)

“I feel there wouldn’t be much point in going to the doctor if you are very sick to be told to wait another few days. I wouldn’t be happy about it.”

(Patient 14)

#### 3.4.3. Symptomatic Treatment for UTI

Two patients had been prescribed symptomatic treatment for UTI in the past. One patient was happy with this treatment as she trusted her GP and the GP was confident it was not a UTI. The other patient indicated she was not adverse to painkillers but she would be less satisfied if symptomatic treatment was recommended as an alternative to antibiotics.

“If it was used as an alternative to antibiotics, I don’t think you would actually be treating the UTI because pain is just a symptom of it so I wouldn’t be happy in that situation. It would affect my decision to return to the GP.”

(Patient 10)

Overall, the patients interviewed expressed uncertainty with accepting symptomatic treatment for a UTI. They were not confident that symptomatic treatment would work as they believed an antibiotic was a necessary treatment for UTI. Some patients would trust their GPs’ recommendations to treat the symptoms, others were sceptical and felt dissatisfied at the thought of treating a UTI with painkillers.

“That’s fine, if they work it’s good…It would influence my decision to return to a GP if it didn’t have an effect on the symptoms.”

(Patient 8)

“Sure a pain killer isn’t going to cure an infection. It will only take the pain away.”

(Patient 14)

## 4. Discussion

To our knowledge this is the first study which explored GP and patient attitudes to delayed antibiotic and symptomatic treatment for UTI. Although the generalisability of the study findings is limited by the qualitative nature of this study and the sample population, this research provides some powerful insights which need to be explored in the future if alternative treatment strategies for UTI were to be promoted. One example of an area that could be further explored is the GP and patients expectations from the consultation. GPs believed that if a patient has a positive dipstick, they need to be treated with an antibiotic and felt the patient expected one. Not all patients participating in this study sought antibiotic treatment, instead wanted relief from symptoms and/or to rule out an infection. However, the findings also revealed that from the patient perspective the appropriateness and acceptability of delayed prescribing may vary based on symptom severity and prior efforts to self-mange before consulting with a GP. It also depended on whether the GP had used a delayed prescribing strategy in the past for UTI. This research also highlighted that there was no exact exact science in the GPs decision to delay antibiotic treatment for UTI. Any combination of reasons or influencers could impact the decision ranging from the GP’s past relationship with the patient (positive and negative influence), patient age or medical card status. Ultimately the GP wanted to ensure the patient did not have to re-consult whilst patients wanted to ensure that they will get back to good health.

Symptomatic treatment only was not used widely to treat UTIs by the GPs who participated in this study. However, GPs said they may recommend symptomatic treatment for patients who have a negative urinalysis and are experiencing vague symptomatic. The GPs were uncomfortable prescribing symptomatic treatment only in case a patient’s symptoms become worse and they had to re-consult. Patients had little experience with symptomatic treatment for a UTI, and therefore were unsure about whether they would be comfortable accepting this treatment. Some seemed to be more willing to accept symptomatic treatment if they also had a delayed prescription.

### 4.1. Comparison with Existing Literature

NICE guidelines recommend “delayed or back up” antibiotic prescriptions and “self care with over the counter preparations” when the infection can be self-limiting. However this research highlights the adoption of these strategies is not as widespread in practice as other infections such as upper respiratory tract infections. GPs will consider using delayed prescribing if they feel a patient has low grade symptoms and have presented too early. Like other research studies investigating GP antibiotic prescribing behaviours [17,18], this research highlighted a multitude of motivators and influencers impacting the GPs decision to prescribe, emphasising the importance of the consultation encounter.

As observed in Leydon et al. (2009) research on UTI patient pathways to the GP [19], the majority of patients in this study had undertaken self care prior to consulting with their GP. However, this was not cited by the patients within this study as a reason not to accept a delay prescription. In another study, thirteen patients were asked to delay taking their prescription and of these thirteen, ten participants gave positive feedback. Only two patients within this study had experienced a delayed prescription, one consumed the antibiotic the other did not.

Despite its use in other areas, patients within this study had minimal experience of delayed prescribing for UTI in the past, suggesting that it is not widely in practice for UTI. However, there was a willingness to accept delayed treatment by half the patients if it had been recommended. These findings are similar to a quantitative study of 176 women, where out of 137 of those who were asked to delay antibiotic treatment, 37% reported that they were happy to accept a delayed prescription. Of those who accepted a delayed prescription, 55% reported not having used the prescription within 7 days [11]. Edwards et al. (2003) found that while two thirds of patients said they had expected an antibiotic at consultation, 92.5% of respondents said they would be happy to accept a delayed prescription in the future [20].

A recent RCT conducted by Gagyor and colleagues showed that the patients recruited to their study were biased towards women with less severe symptom scores [14]. Similarly, our study highlighted that GPs felt that antibiotic treatment was necessary for patients who present with symptoms of a UTI and who have a positive dipstick. Gagyor (2015) recommended that symptomatic treatment should be part of a shared decision making process between the GP and patient with women who are willing to avoid an antibiotic or accept a delayed prescription [14].

### 4.2. Strengths and Limitations

By conducting GP and patient interviews, this research adopted a holistic approach to understanding the factors affecting decisions to issue and accept delayed antibiotic prescriptions and symptomatic treatment for UTI. Qualitative research findings are limited in their generalisability, however, this research contributes to the understanding of treatment strategies and how to overcome barriers. This level of understanding cannot be achieved through quantitative research. Due to the acute nature of the UTI illness a longitudinal study of attitudes and experiences would not have been appropriate.

By interviewing female patients immediately after they consulted with a suspected UTI, patients were very aware of the type of symptoms they experienced and the impact it had on their daily lives. The group of patients were diverse in terms of their age and the length of time they waited before initially consulting the GP with their UTI. However, patient’s attitudes towards delayed prescribing and symptomatic treatment may have been biased by past interactions with their GP.

## 5. Conclusions

This study based in ambulatory care highlights that patients presenting with a suspected UTI are more likely to be treated with an immediate antibiotic than a delay or back-up prescription. Patients are even less likely to receive symptomatic treatment only for UTI symptoms. This research provides insight into why alternative treatments have not been readily adopted into practice. At present there is insufficient evidence to change their treatment behaviours. However, there is scope for changing treatment practices with patients presenting with milder symptoms or who would like to avoid taking antibiotics unnecessarily.

Although not currently widely adopted there was support for delaying treatment for patients who are symptomatic but have a negative dipstick result. The development of guidelines could help GP become more confident in issuing delayed prescriptions for these type of patients but only if further empirical research is conducted in this area highlighting their merit. In general, patients who were given a delayed prescription were verbally advised of the delay, this recommendation could be made more powerful if it was written on prescriptions and followed up and recorded in patient notes.

Overall, GPs were reluctant to recommend symptomatic treatment only as they feared symptoms would worsen and patients would re-consult. As patients are not familiar with symptomatic treatment for UTI, patients may also need additional reassurance that by treating their symptoms with painkillers they will become more comfortable. Further research is needed to demonstrate the effectiveness of symptomatic treatment for UTI before it is adopted into practice or promoted.

Before public health messages or social marketing informed behavioural change interventions can be developed to promote the adoption of alternative treatments for UTI such as delayed and symptomatic treatment of UTI in ambulatory care, rigorous research proving the benefits of these alternative must be undertaken.

## Figures and Tables

**Table 1:** Sample interview questions.

GP Face to Face Interviews	UTI Patient Telephone Interviews
**Section 1:** Antibiotics in general	**Section 1:** Antibiotics in general
Overall, what are your views on prescribing antibiotics? Since you started practicing medicine have your attitudes towards prescribing antibiotics changed? Pros and cons?	Can you describe to me what an antibiotic is? In what sort of circumstances/situations would you expect to be prescribed an antibiotic? Have you ever been prescribed an antibiotic in the past? For what symptoms? How did you feel about receiving the prescription?
**Section 2:** Delayed prescribing in general	**Section 2:** Delayed prescribing
Have you used delayed prescribing before? How long have you been using delayed prescribing? What are your views on this approach? Pros and cons?	Has your GP ever prescribed you an antibiotic, but told you only to fill the prescription if you felt no better, or felt worse after several days? How did you feel about this approach? Can you talk me through how the GP asked you to delay?
**Section 3:** Antibiotic treatment of urinary tract infections	**Section 3:** Treatment of UTI
Can you describe a “typical“ UTI patient? Can you describe each step in your decision making process for treatment of a case like this?	You recently attended your GP with a urinary tract infection, is this correct? Can you describe to me the symptoms you were experiencing at the time of this consultation?
**Section 4:** Symptomatic Treatment	**Section 4:** Experiences of symptomatic treatment
Have you ever used symptomatic treatment for a suspected UTI? Can you describe the pros and cons of symptomatic treatment for a patient with a suspected UTI?	Has your doctor ever given you an antibiotic prescription for a urinary tract infection and told you only to take it if you felt no better or felt worse after a few days? How did you/would you feel about this? Did you/would you follow this advice to delay?

**Table 2:** Example of the themes and sub-themes emerging from analysis.

GP Themes and Sub-Themes	Patient Themes and Sub-Themes
**Delayed prescribing for UTI**	**Delayed treatment of UTI**
Attitude to using delayed treatment in UTI Influences on delaying prescription in UTI Examples of have they used delayed treatment in the past Treatment given Advice provided Delayed prescribing UTI vs. URTI	Experience of a delayed prescription Attitude to delayed prescribing

**Table 3:** Length of time patient waited before visiting the GP.

Days Waited	No. of Patients
First Day	4
2 days	2
3–4 days	3
5–6 days	1
One week	2
>1 week	2
